# Supplementary material for: Lin28, a major translation reprogramming factor, gains access to YB-1-packaged mRNA through its cold-shock domain
Source: Commun Biol. 2021 Mar 19;4:359. doi: 10.1038/s42003-021-01862-3 (PMC7979924; doi:10.1038/s42003-021-01862-3)
Supplement: Supplementary file 2 — Supplementary Information [file 42003_2021_1862_MOESM2_ESM.pdf]

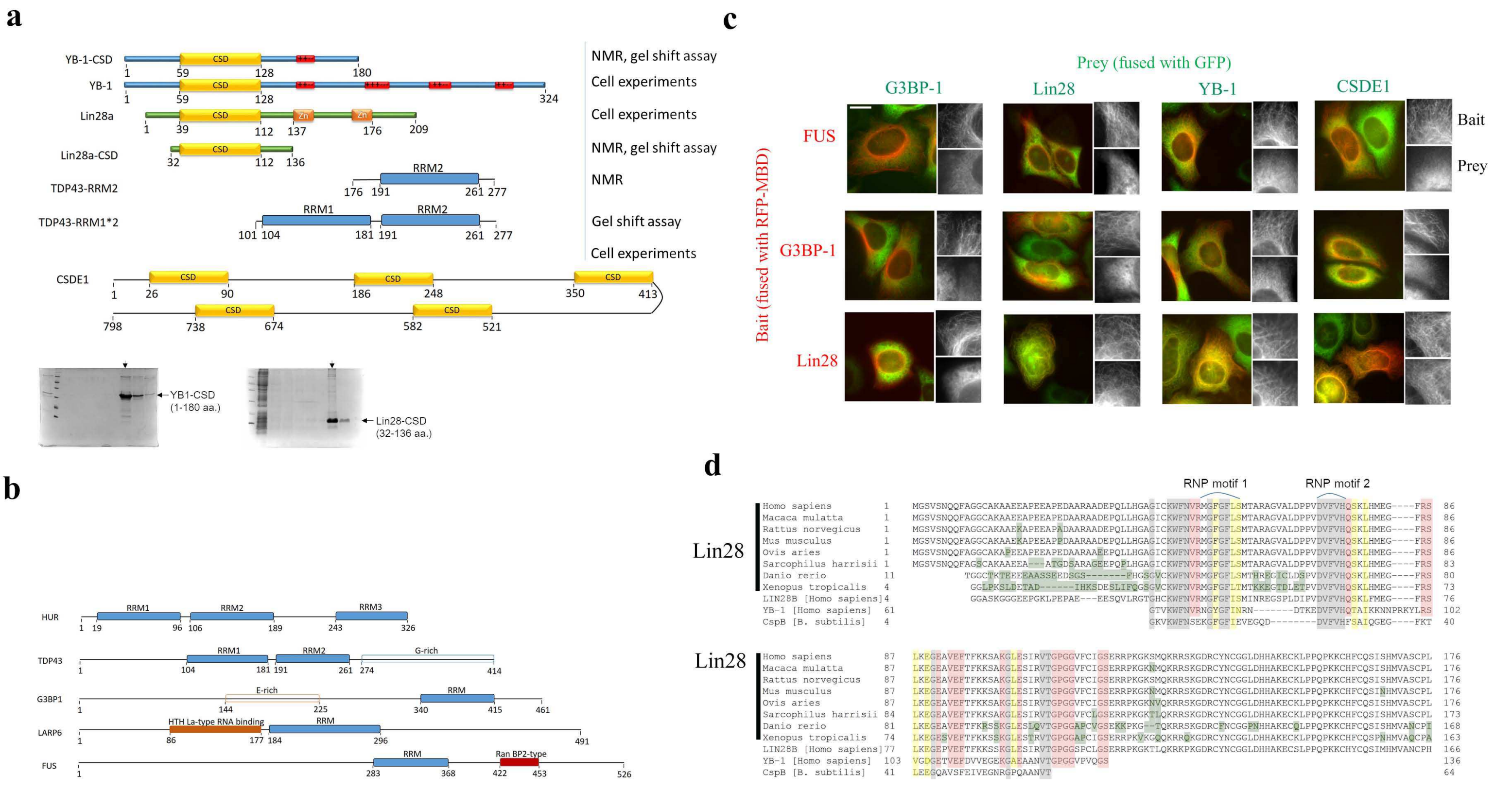

a
YB-1-CSD
YB-1
Lin28a
Lin28a-CSD
TDP43-RRM2
TDP43-RRM1*2
CSDE1
CSD
Zn
RRM1
RRM2
NMR, gel shift assay
Cell experiments
Cell experiments
NMR, gel shift assay
NMR
Gel shift assay
Cell experiments
YB1-CSD (1-180 aa.)
Lin28-CSD (32-136 aa.)
b
HUR
TDP43
G3BP1
LARP6
FUS
RRM1
RRM2
RRM3
G-rich
E-rich
RRM
HTH La-type RNA binding
Ran BP2-type
c
Prey (fused with GFP)
G3BP-1
Lin28
YB-1
CSDE1
Bait (fused with RFP-MBD)
FUS
G3BP-1
Lin28
Bait
Prey
d
RNP motif 1
RNP motif 2
Lin28
Homo sapiens
Macaca mulatta
Rattus norvegicus
Mus musculus
Ovis aries
Sarcophilus harrisii
Danio rerio
Xenopus tropicalis
LIN28B [Homo sapiens]
YB-1 [Homo sapiens]
CspB [B. subtilis]

Figure S1

Supplementary Figure S1: RNA-binding proteins, protein constructs and recombinant proteins considered in the present study.

a. RNA-binding proteins and/or their constructs used in the study. Whether the proteins were considered for NMR analysis, cellular approach (microtubule nanoplatform or expression in cells) and bioinformatics analysis is indicated in the figure. Lower panel: SDS-PAGE gels with the various steps to purify YB-1-CSD and Lin28-CSD.
b. RNA-binding proteins mentioned in the article. RRM is common for many RBPs, unlike cold shock domain.
c. As described in Figure 1a and b, an indicated RBP was used as bait and brought onto the microtubule network in HeLa cells. Then, the interaction of the bait with another RBP used as prey was detected by measuring the presence of the prey on microtubules. Here the representative images are shown for bait/prey localization in HeLa cells corresponding to the data shown in Fig. 1b that were not presented in Fig. 1a. HeLa cells were transfected with plasmids containing (RBP)-RFP-MBD and GFP-(RBP) for 24 h. Scale bar: 15 µm.
d. Alignment of Lin28a orthologue sequences from different species, as well as Lin28b, YB-1 and bacterial cold shock protein B (CspB). In grey, the residues conserved for all these proteins are highlighted; in yellow, conserved type of amino acids; in pink, conserved only in eukaryotes; in green, differences between Lin28A orthologues. Overall, the regions corresponding to RNP motifs 1 and 2 are highly conserved.

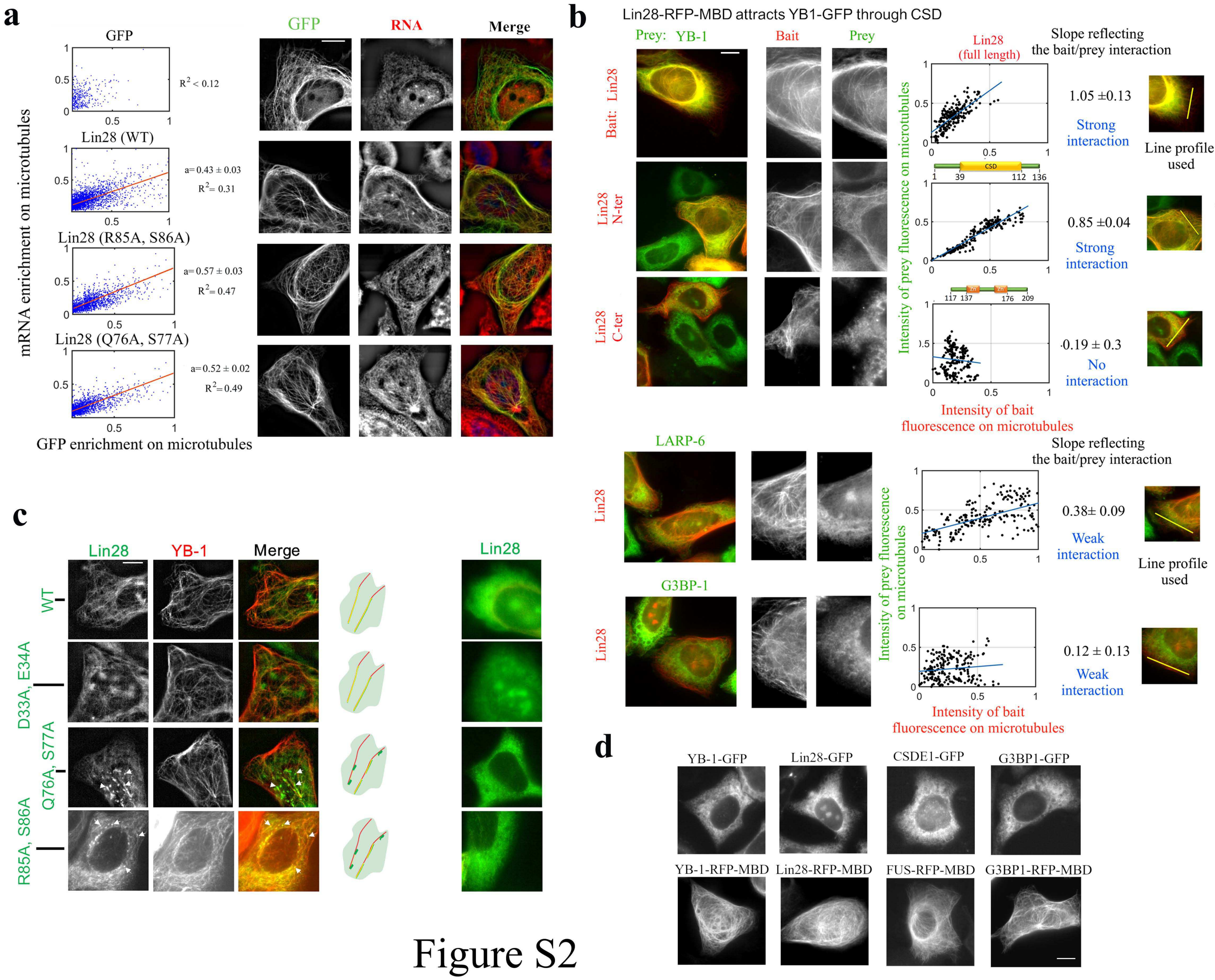
a
GFP
RNA
Merge
Lin28 (WT)
Lin28 (R85A, S86A)
Lin28 (Q76A, S77A)
$R^2 < 0.12$
a= 0.43 ± 0.03
$R^2 = 0.31$
a= 0.57 ± 0.03
$R^2 = 0.47$
a= 0.52 ± 0.02
$R^2 = 0.49$
mRNA enrichment on microtubules
GFP enrichment on microtubules
b
Lin28-RFP-MBD attracts YB1-GFP through CSD
Prey: YB-1
Bait
Prey
Bait: Lin28
Lin28 N-ter
Lin28 C-ter
Lin28 (full length)
Slope reflecting the bait/prey interaction
1.05 ±0.13
Strong interaction
0.85 ±0.04
Strong interaction
-0.19 ± 0.3
No interaction
Line profile used
CSD
Zn
Intensity of prey fluorescence on microtubules
Intensity of bait fluorescence on microtubules
LARP-6
G3BP-1
0.38± 0.09
Weak interaction
0.12 ± 0.13
Weak interaction
c
Lin28
YB-1
Merge
WT
D33A, E34A
Q76A, S77A
R85A, S86A
d
YB-1-GFP
Lin28-GFP
CSDE1-GFP
G3BP1-GFP
YB-1-RFP-MBD
Lin28-RFP-MBD
FUS-RFP-MBD
G3BP1-RFP-MBD

Figure S2

Supplementary figure S2: Lin28 interaction with YB-1 requires the Lin28 cold-shock domain, and Lin28-QS and -RS mutants display an imperfect mixing with YB-1 after a double mutation in the cold-shock domain but still interact with mRNA in cells.

a. Analysis of Lin28 mutant ability to bind mRNA. HeLa cells expressing Lin28-GFP-MBD or GFP-MBD for 24h were fixed. mRNA was detected by in situ hybridization with oligo-dT-[Cy3] (red). Image analysis was performed at the single-cell level using CellProfiler as described in Materials and Methods. Fluorescence intensity was measured on microtubules and in the surrounding cytoplasm. The plotted data represents the mRNA enrichment on microtubules (ratio of microtubule/cytoplasm mean intensity). The slope of the linear fit on the graphs is shown, as well as R^2. The graphs are showing positive correlation between Lin28 and mRNA enrichments on microtubules for wild type and the mutants.
b. Analysis of Lin28-N-ter (aa 1-136) and Lin28-C-ter (aa 117-209) abilities to interact with YB-1. Using the microtubule nanoplatform (Fig. 1a), YB-1 was used as prey and wild type and truncated Lin28 were used successively as baits. Image analysis was performed at the single-cell level to reveal the enrichments of the bait and the preys on microtubules using cross sections. The data were normalized to 1 and plotted as “prey” intensity on microtubules versus “bait” intensity on microtubules. YB-1 used as bait is clearly attracted by wild type Lin28 and Lin28-N-ter, all containing CSD, on microtubules. On the other hand, YB-1 cannot be brought by Lin28-C-ter, containing only the Zinc Fingers domain, on microtubules. In addition, LARP6 and G3BP1 used as control RBPs that bind efficiently to mRNA poorly colocalize with Lin28, used as bait on microtubules. The presence of a CSD domain in the prey RBP is therefore necessary to observe a strong colocalization with YB-1 or Lin28 in cells.
c. Analysis of the ability of wild type and Lin28 mutants to interact with YB-1. Experiments were performed after the expression of YB-1–RFP–MBD and Lin28(WT/mutants)–GFP in HeLa cells for 24h. The colocalization of red (YB-1) and green (Lin28) on microtubules is clearly visible. When Lin28-QS (Q76A, S77A) and Lin28-RS (R85A, S86A) were used as preys, we noticed the presence of small droplets along microtubules (white arrows), in contrast with Lin28(WT) and Lin28-DE (D33A, E34A) that spread along microtubules. We attributed the presence of these droplets to an impaired mixing of Lin28 and YB-1 for Lin28-QS and Lin28-RS. Right panel: In the absence of bait protein expression, wild type and Lin28 mutants are homogenously distributed in the cytoplasm.
d. Representative images of “bait” and “prey” (from Figure 1a,b, Supplementary Figure 1c) distribution in HeLa cells while being overexpressed alone. Preys (YB-1, Lin28, CSDE1, G3BP1 fused to GFP) are homogenously distributed in cytoplasm; baits (YB-1, Lin28, FUS, G3BP1 fused to RFP-MBD) are placed uniformly on the microtubules.
   Scale bars: 15 µm.

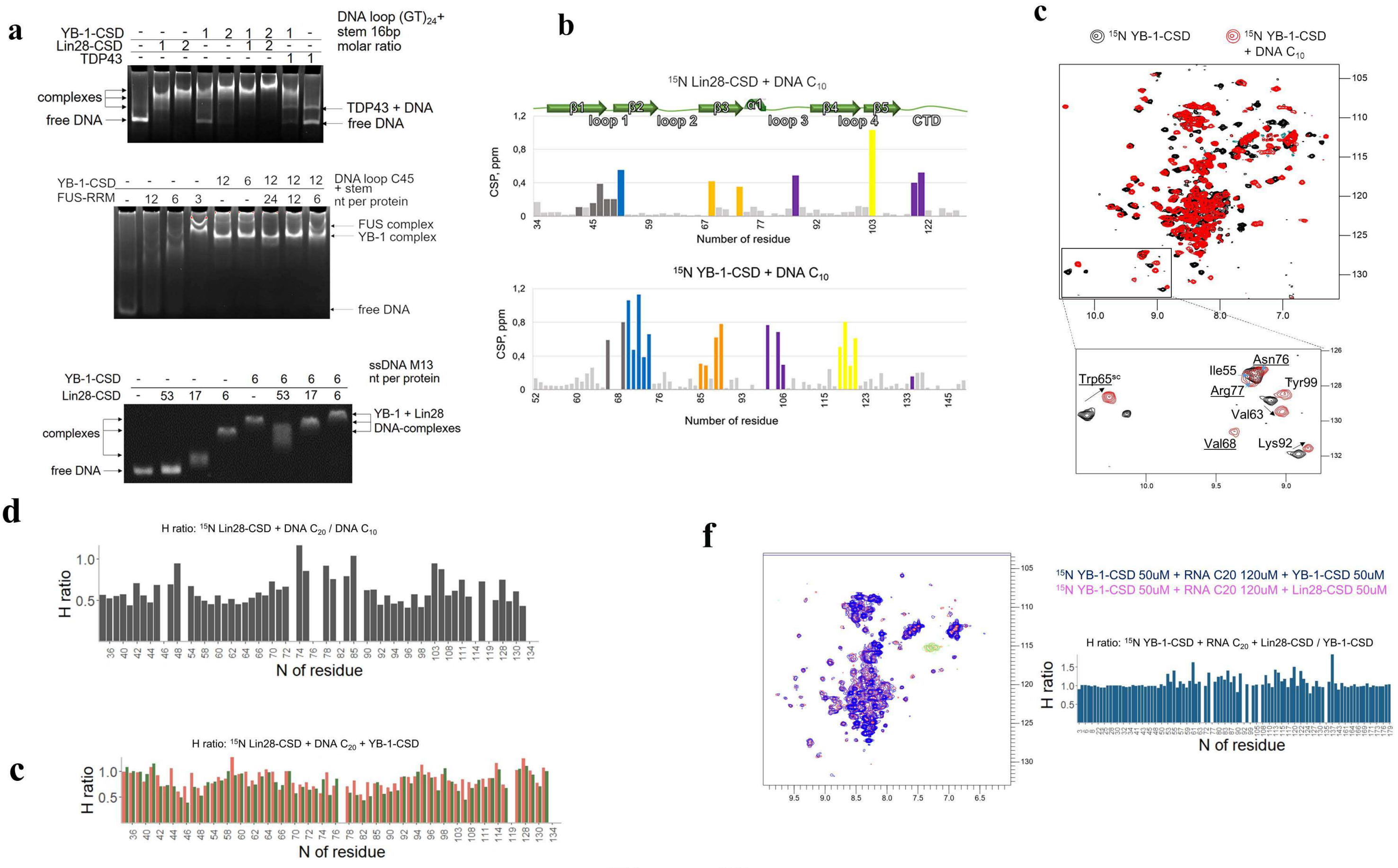
a
YB-1-CSD
Lin28-CSD
TDP43
DNA loop (GT)24+ stem 16bp molar ratio
complexes
free DNA
TDP43 + DNA
free DNA
FUS-RRM
DNA loop C45 + stem nt per protein
FUS complex
YB-1 complex
free DNA
ssDNA M13 nt per protein
complexes
free DNA
YB-1 + Lin28 DNA-complexes
b
15N Lin28-CSD + DNA C10
loop 1
loop 2
loop 3
loop 4
CTD
CSP, ppm
Number of residue
15N YB-1-CSD + DNA C10
CSP, ppm
Number of residue
c
15N YB-1-CSD
15N YB-1-CSD + DNA C10
Trp65sc
Ile55
Asn76
Arg77
Tyr99
Val63
Val68
Lys92
d
H ratio: 15N Lin28-CSD + DNA C20 / DNA C10
H ratio
N of residue
c
H ratio: 15N Lin28-CSD + DNA C20 + YB-1-CSD
H ratio
N of residue
Lin28 + YB-1 2:1
Lin28 + YB-1 1:1
f
15N YB-1-CSD 50uM + RNA C20 120uM + YB-1-CSD 50uM
15N YB-1-CSD 50uM + RNA C20 120uM + Lin28-CSD 50uM
H ratio: 15N YB-1-CSD + RNA C20 + Lin28-CSD / YB-1-CSD
H ratio
N of residue

Figure S3

Supplementary figure S3: YB-1 and Lin28 bind similarly to nucleic acids, and form a heterogenous multimer on ssDNA and RNA *in vitro.*

a. Gel shift assays of TDP-43 RRM 1-2, Lin28-CSD and YB-1-CSD binding to a loop $(GT)_{24}$ + stem 16 bp (TDP-43 has a high affinity for GT repeats); Lin28-CSD and YB-1-CSD binding to long ssDNA M13, FUS RRM and YB-1-CSD binding to a loop $(C)_{45}$ + stem 16bp. Staining with EtBr. Choose of DNA sequence was based on the affinity of used RBPs. Lin28-CSD and YB-1-CSD together give one band of DNA:protein complexes, while other RBPs display bands which are separate from the one of YB-1-CSD complex. Proteins were premixed in a buffer solution containing Tris 20 mM, pH 7.6, NaCl 40 mM, DTT 0.5 mM, then DNA was added at room temperature for 30 min.
b. Upper panel: CSPs of Lin28-CSD residues upon binding to 10 nt-long poly(C) oligonucleotide. Lower panel: the same for YB-1-CSD (aa 1-180);
c. NMR spectra of YB-1-CSD interaction with DNA C_{10}. ^{1}H-^{15}N HSQC spectra and corresponding zoom in on peaks of indicated residues including RNA-binding residues (underlined) (black – free protein, red – bound state to DNA C_{10}). . T = 25°C, K-phosphate buffer 50 mM, pH 6.8, 512 scans.
d. NMR peak intensity variations from ^{1}H-^{15}N HSQC spectra of Lin28-CSD in the presence of 20 nt versus 10 nt-long ssDNA Poly(C)) (spectra not shown). A decrease in peak intensity reflects the presence of multiple proteins on the 20 nt-long ss DNA C_{20} while a single Lin28-CSD interacts with 10 nt-long ssDNA. T = 30°C, K-phosphate buffer 50 mM, pH 7, NaCl 250 mM, 512 scans.
e. NMR peak height analysis from ^{1}H-^{15}N HSQC spectra of Lin28-CSD in the presence of ssDNA (20 nt-long Poly(C)) upon addition of non-labeled YB-1-CSD at increasing concentrations. A progressive decrease in peak intensity should be observed when the molecular weight of the DNA:protein complex increases owing to a decrease in mobility. T = 30°C, K-phosphate buffer 50 mM, pH 7, NaCl 300 mM, 512 scans. Proteins were first mixed, then DNA was added.
f. NMR analysis from ^{1}H-^{15}N HSQC spectra of YB-1-CSD in the presence of RNA (20 nt-long Poly(C)) and non-labeled YB-1-CSD (blue) or Lin28-CSD (pink). Histogram reflecting peak height ratio of NMR peaks from ^{1}H-^{15}N HSQC spectra under indicated conditions. T = 30°C, K-phosphate buffer 50 mM, pH 7, NaCl 300 mM, 256 scans; proteins were first mixed, then RNA was added.

a

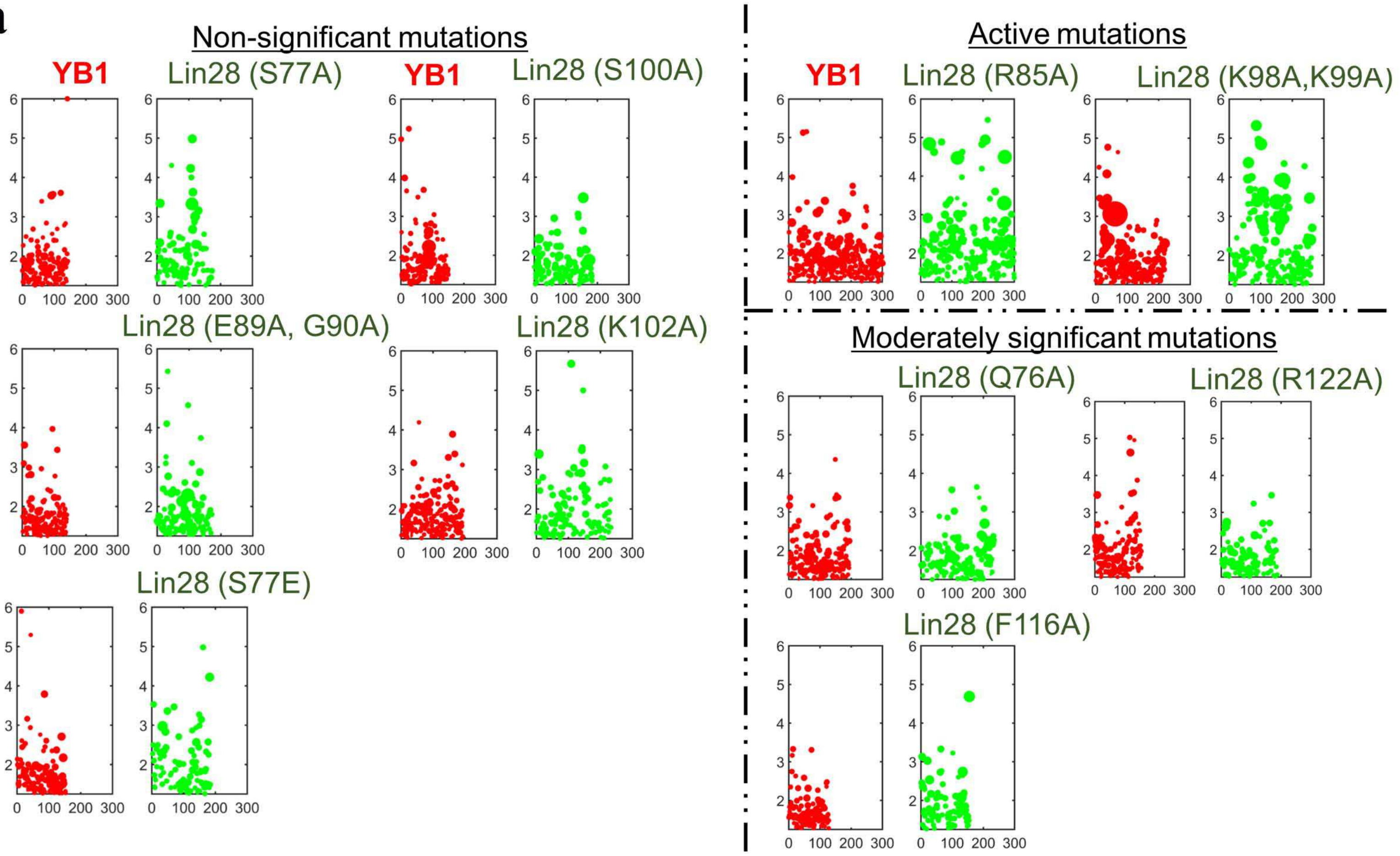

b

	t-test						Kolmogorov–Smirnov test				
						Mutation					
	To ctrl	To S86A	To F55A	To H80A	To G114A,V115A		To ctrl	To S86A	To F55A	To H80A	To G114A,V115A
Control & Passive mutations	1,00E+00	8,74E-01	1,71E-01	4,27E-02	6,50E-02	CTRL	1,00E+00	7,88E-01	6,17E-01	4,77E-01	7,15E-01
	8,74E-01	1,00E+00	2,67E-01	8,55E-02	1,08E-01	S86A	7,88E-01	1,00E+00	5,79E-01	7,08E-01	6,85E-01
	1,71E-01	2,67E-01	1,00E+00	5,36E-01	3,68E-01	F55A	6,17E-01	5,79E-01	1,00E+00	3,49E-01	9,76E-01
	4,27E-02	8,55E-02	5,36E-01	1,00E+00	5,73E-01	H80A	4,77E-01	7,08E-01	3,49E-01	1,00E+00	4,89E-01
	6,50E-02	1,08E-01	3,68E-01	5,73E-01	1,00E+00	G114A, V115A	7,15E-01	6,85E-01	9,76E-01	4,89E-01	1,00E+00
Non-significant mutations	8,48E-03	1,79E-02	6,23E-02	6,61E-02	1,75E-01	S77A	1,67E-02	6,15E-02	5,03E-01	1,69E-01	2,06E-01
	4,84E-03	1,20E-02	7,02E-02	1,06E-01	3,60E-01	S100A	7,61E-02	2,01E-01	5,69E-01	3,09E-02	3,20E-01
	1,37E-03	5,08E-03	9,61E-02	2,67E-01	8,72E-01	K102A	7,07E-02	1,91E-01	7,29E-01	2,24E-01	5,13E-01
	3,83E-04	1,74E-03	3,92E-02	1,25E-01	6,22E-01	E89A,G90A	2,31E-01	1,71E-01	5,77E-01	3,70E-01	3,91E-01
	2,94E-04	1,41E-03	3,59E-02	1,14E-01	5,99E-01	S77E	2,77E-02	9,07E-02	5,22E-01	3,20E-01	3,02E-01
Moderately significant	4,96E-03	1,18E-02	5,64E-02	7,06E-02	2,27E-01	R122A	2,13E-02	5,71E-02	1,88E-01	9,13E-03	1,66E-01
	2,62E-04	1,37E-03	5,01E-02	1,80E-01	7,91E-01	Q76A	5,09E-03	1,56E-02	2,08E-01	8,29E-02	7,79E-02
	1,53E-05	1,16E-04	4,78E-03	1,48E-02	2,18E-01	F116A	3,91E-03	1,67E-02	2,00E-01	2,45E-01	7,96E-02
Significant mutations	8,20E-06	6,97E-05	3,76E-03	9,97E-03	1,52E-01	R85A,S86A	1,39E-03	5,60E-03	6,29E-02	2,56E-03	2,92E-02
	1,03E-05	6,11E-05	5,63E-04	2,50E-04	1,56E-03	K98A,K99A	2,25E-14	4,27E-13	1,46E-09	1,33E-10	1,98E-09
	4,52E-06	3,72E-05	1,20E-03	1,55E-03	2,34E-02	Q76A,S77A	5,44E-05	7,12E-04	3,77E-02	1,48E-03	9,94E-03
	2,33E-07	3,51E-06	3,56E-04	7,67E-04	3,13E-02	R122A,R123A	1,40E-04	1,40E-04	8,51E-03	1,23E-04	4,57E-03
	2,01E-07	2,95E-06	2,42E-04	3,94E-04	1,57E-02	G119A,S120A	1,57E-06	4,15E-06	8,39E-04	2,32E-05	2,89E-04
	5,91E-09	1,33E-07	9,19E-06	3,97E-06	1,41E-04	R85A	1,24E-21	7,74E-18	3,85E-12	2,20E-13	4,78E-14
	1,25E-08	2,26E-07	7,97E-06	2,07E-06	4,07E-05	F47A	3,70E-17	2,89E-15	4,68E-11	1,45E-12	1,58E-11

Significant difference: P-value < 0,05

Non-significant difference: P-value ≥ 0,05

Significant demix according to ≥ 4 [control and passive mutations] in KS test

Significant demix according to ≥ 2 [control and passive mutations] in KS test

Non-significant in KS test

Figure S4

Supplementary figure S4: Analysis of the mixing/demixing between YB-1 and Lin28 (WT/mutants) along the microtubule network.

a. Representative images of indicated mixing/demixing Lin28 mutants and YB-1 along microtubules in HeLa cells. Plot of the length and enrichment of RFP or GFP compartments measured along the microtubule network (>7 mm per condition). The obtained data for every Lin28 mutation were plotted on the graphs as relative enrichment ratio in compartment versus compartment number. Sphere radii in the graphs are proportional to square root of length of the corresponding clusters. ** $p < 0.01$, Student's test with two tails compared with control Lin28 proteins.
b. Statistical analysis of mixing/demixing between YB-1 and Lin28 (WT/mutants). We used a t-test with two tails and a Kolmogorov-Smirnov test (ks test) to assess whether the enrichment of two different Lin28 mutants in compartments were significantly different. We found that wild type Lin28 and 4 Lin28 mutants (passive mutation) did not display a significantly different enrichment in compartments detected on microtubules. Significance of the enrichment in compartments was then determined relatively to the wild type Lin28 and to the passive mutations with the corresponding P-values for the t- and ks tests. (green: significantly different, yellow: not different).

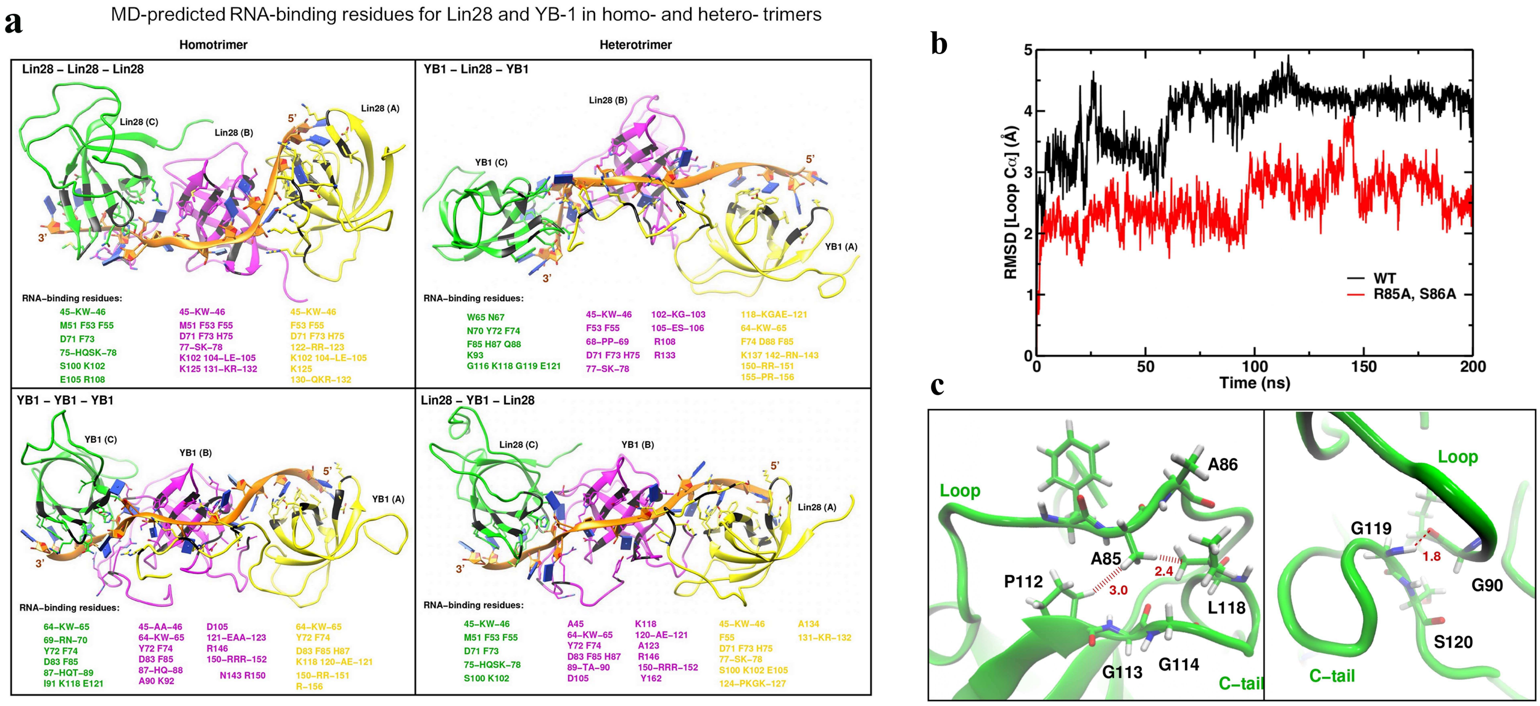
a
MD-predicted RNA-binding residues for Lin28 and YB-1 in homo- and hetero- trimers
Homotrimer
Heterotrimer
Lin28 – Lin28 – Lin28
YB1 – Lin28 – YB1
YB1 – YB1 – YB1
Lin28 – YB1 – Lin28
RNA–binding residues:
b
RMSD [Loop Cα] (Å)
Time (ns)
WT
R85A, S86A
c
Loop
C–tail
A86
A85
P112
L118
G113
G114
G119
G90
S120
3.0
2.4
1.8

Figure S5

Supplementary figure S5: Modeling of Lin28 and YB-1 homo- and heterotrimers in presence of 16 nt-long ssRNA by molecular dynamics.

a. All the trimers appeared to be stable (see Materials and Methods for details). Residues that participate to RNA binding are represented in blue and listed at the bottom of each panel.
b. We monitored the conformational changes of the Lin28 loop 3 in the YB-1/Lin28/YB-1 trimer for the WT and R85A/S86A mutant. The C_alpha RMSD of Lin28 loop 3 is represented. We observed that the movements of the loop are reduced when R85/S86 are mutated into alanine residues.
c. Structure of Lin28 R85A/S86A in the YB-1/Lin28/YB-1 trimer focusing on specific interactions between the loop 3 and CTD. The left and right panels illustrate interactions engaging A85 and G119, respectively. The red dashed line indicates a hydrogen bond, and the enlarged red dashed lines indicate hydrophobic interactions. The distances in Angstrom are also reported.

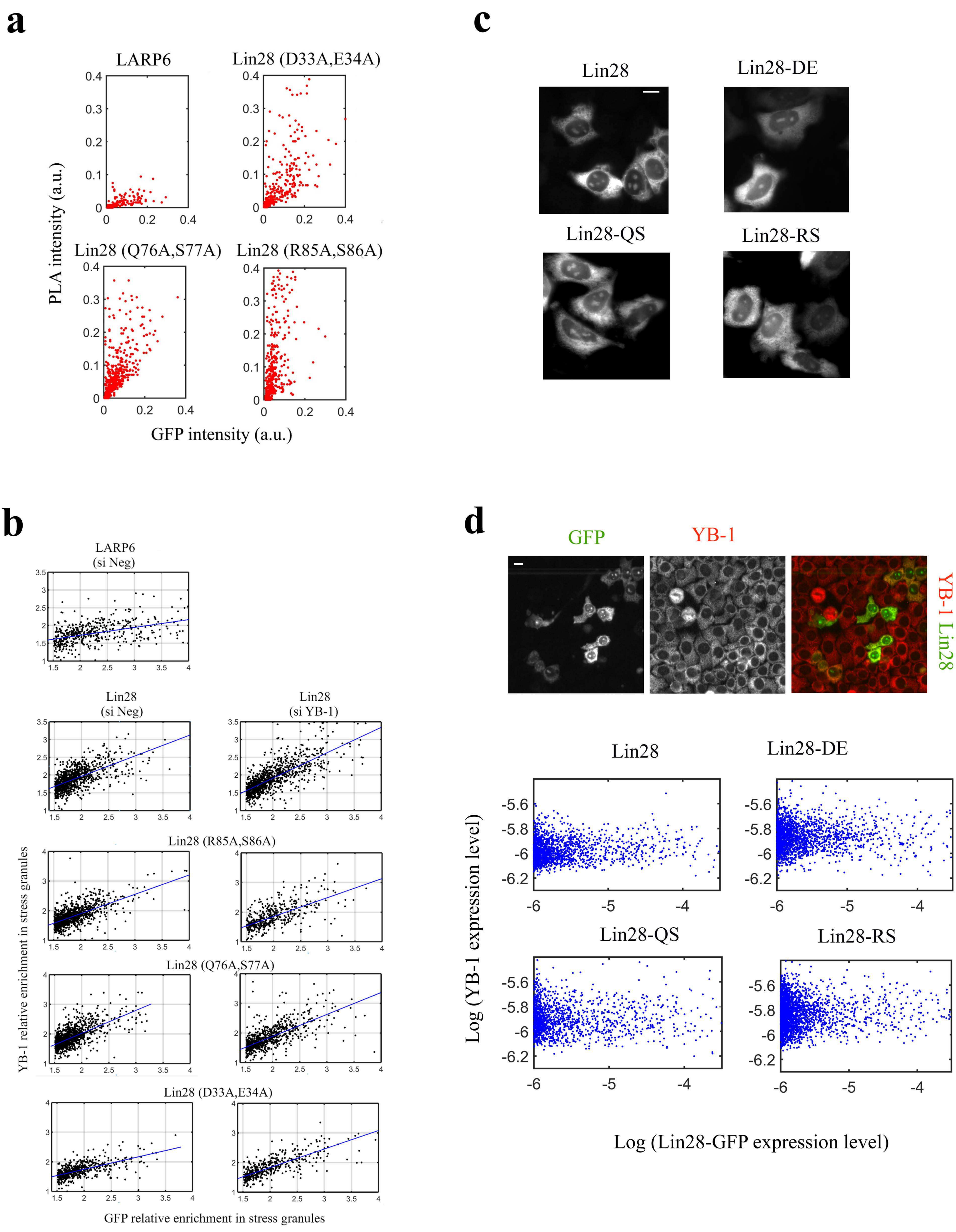
a
LARP6
Lin28 (D33A,E34A)
Lin28 (Q76A,S77A)
Lin28 (R85A,S86A)
PLA intensity (a.u.)
GFP intensity (a.u.)
b
LARP6
(si Neg)
Lin28
(si Neg)
Lin28
(si YB-1)
Lin28 (R85A,S86A)
Lin28 (Q76A,S77A)
Lin28 (D33A,E34A)
YB-1 relative enrichment in stress granules
GFP relative enrichment in stress granules
c
Lin28
Lin28-DE
Lin28-QS
Lin28-RS
d
GFP
YB-1
YB-1 Lin28
Lin28
Lin28-DE
Lin28-QS
Lin28-RS
Log (YB-1 expression level)
Log (Lin28-GFP expression level)

Figure S6

Supplementary figure S6: YB-1 interactions with wild type and mutant Lin28.

a. Proximity ligation assay (PLA) performed for YB-1 and Lin28a mutants, LARP6 as control. The plasmids with Lin28/LARP6–GFP were used for HeLa transfection. After 24h of incubation the cells were fixed and the antibodies against YB-1 and GFP were applied, following with conventional PLA protocol (see Materials and Methods). PLA intensity was measured by red probe fluorescence using CellProfiler and plotted on graphs versus GFP intensity. The increase of PLA signal is observed for Lin28 (wt and mutants) showing the colocalization with YB-1, unlike for LARP6.
b. Colocalization of endogenous YB-1 and wild type and Lin28 mutants in stress granules (SGs) with and without decreasing endogenous YB-1 levels in HeLa cells. HeLa cells expressing Lin28-GFP were exposed to 300 µM arsenite for 1 h at 37°C, then fixed. Anti-YB-1(red). Stress granules in cells were detected automatically using CellProfiler. The mean fluorescence intensities of YB-1 and Lin28 in the stress granules and in the surrounding cytoplasm were then measured. The relative YB-1 versus Lin28-GFP enrichments in stress granules are represented in a 2D plot to measure the slope. More than 400 stress granules were detected for each condition. As control, we measured the relative enrichment of endogenous YB-1 in stress granules in HeLa cells expressing LARP6-GFP. According to the less pronounced slope, the relative enrichment of endogenous YB-1 is less significant in LARP6- than Lin28-rich stress granules. Scale bar: 15 µm.
c. The subcellular localization of Lin28 in cells is not changed upon indicated mutations. Lin28 is mostly homogenously distributed in the cytoplasm but is also present in the nucleoli.
d. Upper panel: coexpression of Lin28 and YB-1 in HeLa cells does not change the subcellular location of the two proteins, both being present in cytoplasm. Lower panel: Expression level of endogenous YB-1 does not vary upon the expression of wild type and Lin28 mutants. HeLa cells expressing GFP-labeled wild type or Lin28-GFP mutants for 72h were fixed, then, endogenous YB-1 was detected with an anti-YB-1 antibody. Lin28 and YB-1 expression levels were estimated at single cell level by measuring the intensity for GFP (for wild type or Lin28 mutants) and Alexa594 (for anti-YB-1 antibody). Scale bar: 15 µm.

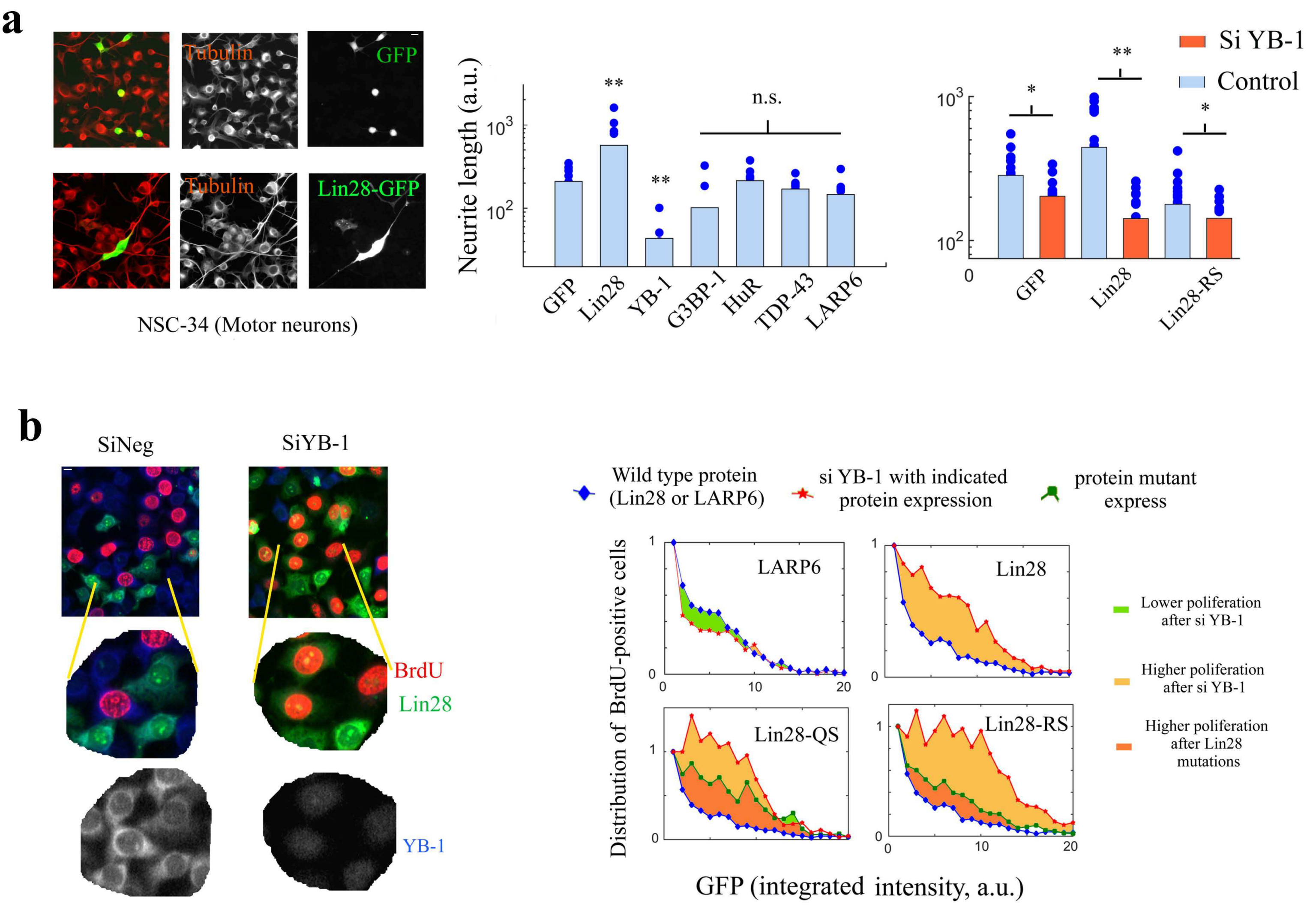
a
Tubulin
GFP
Tubulin
Lin28-GFP
NSC-34 (Motor neurons)
Neurite length (a.u.)
n.s.
**
**
GFP
Lin28
YB-1
G3BP-1
HuR
TDP-43
LARP6
Si YB-1
Control
*
**
*
GFP
Lin28
Lin28-RS
b
SiNeg
SiYB-1
BrdU
Lin28
YB-1
Wild type protein (Lin28 or LARP6)
si YB-1 with indicated protein expression
protein mutant express
Distribution of BrdU-positive cells
LARP6
Lin28
Lin28-QS
Lin28-RS
Lower poliferation after si YB-1
Higher poliferation after si YB-1
Higher poliferation after Lin28 mutations
GFP (integrated intensity, a.u.)

Figure S7

Supplementary figure S7: Lin28 promotes a YB-1-dependent extension of neurites; Lin28 regulates cell proliferation in a YB-1-dependent manner.

a. Representative images showing the proliferation status of HeLa cells expressing Lin28-GFP for 72 h and treated with YB-1 siRNA or control siRNA. BrdU staining (proliferative cells, red nucleus). Analysis at the single cell level, BrdU-positive cells were recorded and classified according to the expression level of the indicated GFP-labeled proteins. The number of BrdU positive cells was normalized to 1 for the zero-expression level of indicated GFP-labeled proteins. Scale bar: 15 µm.
b. Left panel: Retinoic acid differentiated NSC-34 motor neurons expressing GFP or Lin28-GFP. Right panel: Measurement of neurite lengths in motor neurons expressing indicated GFP-labeled RBPs with or without decreasing YB-1 levels with siRNA. ** $p < 0.01$, t-test with two tails. $n > 15$. Scale bar: 15 µm.

Table S1

Molecule B

Lin28 - **Lin28** - Lin28	P112	G113	G114	V115	F116	C117	**I118**	**G119**	**S120**	E121	R122	R123	P124	**Total CTD**
L79	-0.03	0	0	0	0	0	0	0	0	0	0	0	0	-0.03
H80	-5.01	-0.05	0	0	0	0	-0.01	0	0	0	0	0	0	-5.07
M81	-0.07	0	0	0	0	0	0	0	0	0	0	0	0	-0.07
E82	-0.01	0	0	0	0	0	0	0	0	0	0	0	0	-0.01
G83	-0.26	0	0	0	0	0	0	0	0	0	0	0	0	-0.26
F84	-9.88	-0.07	-0.08	-0.01	0	0	-0.07	0	0	0	0	0	0	-10.12
R85	-5.05	-0.67	-0.666	0	0	0	-0.39	0	0	0	0	0	0	-6.77
**S86**	-8.57	-16.6	-12.8	-8.35	-0.02	-0.07	-6.42	-0.41	-0.01	0	0	0	0	**-53.31**
L87	-0.2	-0.01	-0.05	0	0	-0.05	-6.98	-0.41	0	0	0	0	0	-7.7
K88	0	0	-0.01	0	-0.01	-0.4	-7.54	-0.5	-0.01	0	0	0	0	-8.47
**E89**	0	0	-0.01	-0.01	-0.29	-12.35	-21.59	-21.53	-59.06	-0.05	-0.08	0	0	**-114.97**
G90	-0.01	-0.01	-0.02	0	-0.05	-0.57	-4.98	-5.53	-5.94	-0.04	0	0	0	-17.14
E91	-0.55	-0.01	-0.14	-0.01	-0.12	-0.18	-4.86	-3.93	-1.26	-0.07	0	0	0	-11.13
**A92**	-9.21	-15.05	-3.38	-0.23	-1.49	-0.33	-4.9	-7.08	-0.75	-0.19	0	0	0	**-42.6**
**Total Loop 3**	-38.86	-32.51	-17.15	-8.6	-1.98	-13.94	**-57.74**	**-39.38**	**-67.04**	-0.36	-0.08	0	0	-277.64

YB-1 - **Lin28** - YB-1	P112	G113	G114	V115	**F116**	C117	**I118**	G119	S120	E121	**R122**	R123	P124	**Total CTD**
L79	0	0	0	0	0	0	0	0	0	0	0	0	0	0
H80	0	0	0	0	0	0	-0.23	0	0	0	0	0	0	-0.23
M81	0	0	0	0	0	0	0	0	0	0	0	0	0	0
E82	0	0	0	0	0	0	0	0	0	0	0	0	0	0
G83	0	0	0	0	0	0	0	0	0	0	0	0	0	0
F84	-0.05	0	0	0	0	0	-0.07	-0.06	0	0	0	0	0	-0.18
R85	0	0	0	0	0	0	-0.04	0	0	0	0	0	0	-0.04
S86	0	0	0	0	0	-0.62	0.21	-0.08	0	0	0	0	0	-0.5
L87	-0.28	-0.06	0	0	-0.01	-1.38	-22.58	-3.38	-0.18	-0.01	0	0	0	-27.88
**K88**	-0.06	-0.17	-0.11	-0.06	-32.94	-8.04	-14.88	-11.15	-5.41	-0.09	-0.06	0	0	**-72.97**
**E89**	-0.17	-0.04	0	0	-0.02	-0.25	-4.69	-9.96	-1.05	-0.61	-84.02	-0.16	0	**-100.96**
G90	-0.22	0	0	0	0	0	-0.15	-0.78	-0.03	-0.04	-0.07	-0.01	0	-1.32
**E91**	-0.2	0	0	0	0	0	-0.01	-0.07	0	-0.1	-66.87	-0.65	-0.07	**-67.97**
A92	-4.17	-0.06	0	0	0	0	-0.07	-0.76	-0.14	-4.49	-3.79	-5.39	-0.31	-19.17
**Total Loop 3**	-5.14	-0.33	-0.11	-0.06	**-32.98**	-10.29	**-42.52**	-26.24	-6.81	-5.34	**-154.81**	-6.22	-0.38	-291.22

YB-1 - **YB1** - YB-1	T126	G127	P128	G129	G130	V131	P132	V133	**Q134**	**G135**	**S136**	K137	Y138	A139	A140	D141	R142	N143	**Total CTD**
I91	-1.79	-0.48	-0.02	0	0	0	0	0	0	0	0	0	0	0	0	0	0	0	-2.29
K92	-1.66	-0.18	0	0	0	0	0	0	0	0	0	0	0	0	0	0	0	0	-1.84
K93	-11.86	-1.57	-0.28	0	0	0	0	0	0	0	0	0	0	0	0	0	0	0	-13.71
N94	0.05	-2.6	-0.94	-0.01	0	0	0	0	-0.02	0	0	0	0	0	0	0	0	0	-3.51
N95	-0.59	-2.83	-7.93	-0.11	0	0	0	0	-1.49	-0.07	0	0	0	0	0	0	0	0	-13.03
P96	-0.32	-0.43	-2.96	-0.25	-0.02	-0.05	0	0	-1.29	-0.01	0	0	0	0	0	0	0	0	-5.32
R97	0	0	-0.03	0	0	0	0	0	-0.09	0	0	0	0	0	0	0	0	0	-0.12
K98	0	0	-0.05	0	0	-0.2	0	-0.04	-0.23	-0.02	0	0	0	0	0	0	0	0	-0.54
**Y99**	0	-0.06	-2.11	-0.3	-0.04	-0.14	-0.01	-0.77	-23.76	-7.65	-1.46	-0.03	0	0	0	0	0	0	**-36.32**
**L100**	0	0	-0.04	-0.02	-0.03	-0.77	-0.39	-9.41	-28.82	-0.69	-0.2	-0.17	0	0	0	0	0	0	**-40.54**
**R101**	0	0	-0.08	-0.01	0	-0.02	-0.07	-6.49	-15.25	-17.65	-24.82	-9.89	-1.13	-0.05	0	0	0	0	**-75.44**
S102	0	0	-0.03	0	0	0	0	-0.35	0.1	-1.09	-0.64	-1.27	-0.05	-0.01	0	0	0	0	-3.34
V103	0	0	0	0	0	0	0	-0.15	-0.03	-0.39	-6.08	-5.83	-2.12	-1.26	-0.23	-0.02	0	0	-16.09
G104	0	0	0	0	0	0	0	0	0	0	-0.15	-0.03	-0.01	-0.02	0	0	0	0	-0.21
D105	0	0	0	0	0	0	0	0	0	0	0	0	0	0	0	0	0	0	0
G106	0	0	-0.01	0	0	0	0	0	0	-0.07	-0.28	0	0	0	0	0	0	0	-0.36
**Total Loop 3**	-16.17	-8.16	-14.47	-0.69	-0.1	-1.19	-0.47	-17.2	**-70.87**	**-27.63**	**-33.62**	-17.21	-3.31	-1.34	-0.23	-0.02	-3.00E-05	0	-212.68

Lin28-**YB1**-Lin28	T126	G127	**P128**	G129	G130	V131	P132	V133	**Q134**	G135	**S136**	K137	Y138	A139	A140	D141	R142	N143	**Total CTD**
I91	-1.66	-0.12	0	0	0	0	0	0	0	0	0	0	0	0	0	0	0	0	-1.78168
K92	-5.13	-0.08	0	0	0	0	0	0	0	0	0	0	0	0	0	0	0	0	-5.2095
K93	-8.34	-1.27	-0.01	0	0	0	0	0	0	0	0	0	0	0	0	0	0	0	-9.62401
N94	-0.62	-0.68	-0.05	-0.01	0	0	0	0	0	0	0	0	0	0	0	0	0	0	-1.3554
N95	-1.15	-10.33	-3.25	-0.17	-0.02	0	0	0	-0.15	-0.05	0	0	0	0	0	0	0	0	-15.1168
P96	-0.52	-4.54	-3.5	-2.76	-0.32	0	0	0	-0.16	-0.01	0	0	0	0	0	0	0	0	-11.8144
R97	0	-0.04	-0.11	-0.07	-0.06	0	0	0	-0.04	0	0	0	0	0	0	0	0	0	-0.32892
K98	0	-0.05	-1.71	-0.95	-2.57	-4.07	-0.11	-0.27	-3.33	-0.09	0	0	0	0	0	0	0	0	-13.1475
Y99	-0.03	-0.73	-10.06	-1.4	-0.89	-0.21	-0.04	-0.55	-13.46	2.89	-1.25	-0.03	0	0	0	0	0	0	-25.7627
L100	0	0	-0.33	-0.04	-0.39	-0.4	-0.09	-3.65	-20.99	-1.56	-0.13	-0.23	0	0	0	0	0	0	-27.8128
**R101**	0	0	-0.25	0	-0.01	-0.03	-0.06	-7.69	-17.5	-17.81	-26.02	-6.98	-0.15	0	0	0	0	0	**-76.5092**
**S102**	0	0	-0.09	0	0	0	0	-0.37	-0.1	-0.72	-30.75	-2.39	-0.15	-0.04	0	0	0	0	**-34.6191**
**V103**	0	0	0	0	0	0	0	-0.14	-0.01	-0.01	-27.83	-8.52	-4.38	-4.04	-0.11	-0.02	0	0	**-45.0599**
G104	0	0	0	0	0	0	0	0	0	0	-0.07	-0.06	-0.02	-0.04	0	0	0	0	-0.19128
D105	0	0	0	0	0	0	0	0	0	0	-0.01	0	0	0	0	0	0	0	-0.01308
G106	0	0	-0.02	0	0	0	0	0	0	-0.05	-0.05	0	0	0	0	0	0	0	-0.11323
**Total Loop 3**	-17.45	-17.8	**-19.38**	-5.39	-4.261	-4.71	-0.318	-12.67	**-55.7**	-17.4107	**-86.12**	-18.2	-4.7	-4.12111	-0.1	-0.018	0	0	-268.46

Supplementary Table 1. MD-based interaction energy calculation between residues of loop 3 and residues from the beginning of CTD, for Lin28 and YB-1 in position B, in the modelled homo- and hetero-trimers. Energies were averaged over 200 ns of MD simulation and values are reported in kJ/mol with variant of fluctuations being ± 0.4 kJ/mol. The most contributing residues are highlighted in bold. The red boxes show the conserved residues that have the highest impact while mutated.
