# Supplementary material for: Lin28, a major translation reprogramming factor, gains access to YB-1-packaged mRNA through its cold-shock domain
Source: Commun Biol. 2021 Mar 19;4:359. doi: 10.1038/s42003-021-01862-3 (PMC7979924; doi:10.1038/s42003-021-01862-3)
Supplement: Supplementary file 3 — Description of Additional Supplementary Files [file 42003_2021_1862_MOESM3_ESM.pdf]

Description of Additional Supplementary Files

File Name: Supplementary Data 1

Description: Fig. 1b- Data represented in figure 1b. Prey and Bait fluorescence intensity in the cytoplasm of HeLa cells versus the Spearman's correlation score measured at the single cell level are shown as described in Methods. The interaction score is the spearman coefficient obtained at zero bait expression level obtained by using a linear regression. The error interval is given with 95 % confidence bounds.

Fig1.d- Raw data shown in Fig. 1d. Intensity of the PLA signal are indicated with the corresponding GFP fluorescence level at the single cell level. The data are presented in HeLa cells expressing Lin28-GFP, LARP6-GFP or GFP alone.

File Name: Supplementary Data 2

Description: NMR data shown in this article including CSPs, peak heights and height ratios.

File Name: Supplementary Data 3

Description: Raw data obtained with the "microtubule bench" assay to measure the mixing/demixing of wild type or Mutant Lin28 with wild type YB-1. RFP and GFP fluorescence intensity along microtubules are shown in cells expressing YB-1-GFP-MBD and wild type or mutant Lin28-RFP-MBD as indicated. The analysis at the raw data is described in the legend of Fig. 4 and Methods.

File Name: Supplementary Data 4

Description: Fig. 6b- Data shown in Fig. 6b. Mean intensity of indicated GFP-labelled proteins (Lin28, G3BP-1, LARP6) and wild type YB-1 (anti-YB-1 antibody) in stress granules. The relative GFP/YB-1 enrichment is the ratio of the mean intensity inside and outside stress granules.

Fig. 6d- RT-PCR data plotted in Fig. 6d.

File Name: Supplementary Data 5

Description: Raw data used to obtain the SG/cytoplasm enrichment of YB-1 and indicated GFP-tagged proteins (Figure 6d and Supplementary Figure S6). The enrichment is the ratio of the mean stress granule/cytoplasm intensity. Raw data were then analyzed by using a linear regression. The results shown in the histogram of Fig. 6d are the values of the slope with a 95% confidence bound.

File Name: Supplementary Data 6

Description: Expression levels of abundant RBPs and Lin28 collected from the Human Protein Atlas and used to measure the correlation score presented in Fig. 7a.
