# Supplementary material for: Lin28, a major translation reprogramming factor, gains access to YB-1-packaged mRNA through its cold-shock domain
Source: Commun Biol. 2021 Mar 19;4:359. doi: 10.1038/s42003-021-01862-3 (PMC7979924; doi:10.1038/s42003-021-01862-3)
Supplement: Supplementary file 10 — Reporting Summary [file 42003_2021_1862_MOESM10_ESM.pdf]

nature research

Corresponding author(s): David pastre

Last updated by author(s): Feb 9, 2021

Reporting Summary

Nature Research wishes to improve the reproducibility of the work that we publish. This form provides structure for consistency and transparency in reporting. For further information on Nature Research policies, see our Editorial Policies and the Editorial Policy Checklist.

Statistics

For all statistical analyses, confirm that the following items are present in the figure legend, table legend, main text, or Methods section.

n/a	Confirmed	
☐	☒	The exact sample size (*n*) for each experimental group/condition, given as a discrete number and unit of measurement
☐	☒	A statement on whether measurements were taken from distinct samples or whether the same sample was measured repeatedly
☐	☒	The statistical test(s) used AND whether they are one- or two-sided<br>*Only common tests should be described solely by name; describe more complex techniques in the Methods section.*
☒	☐	A description of all covariates tested
☒	☐	A description of any assumptions or corrections, such as tests of normality and adjustment for multiple comparisons
☐	☒	A full description of the statistical parameters including central tendency (e.g. means) or other basic estimates (e.g. regression coefficient) AND variation (e.g. standard deviation) or associated estimates of uncertainty (e.g. confidence intervals)
☐	☒	For null hypothesis testing, the test statistic (e.g. *F*, *t*, *r*) with confidence intervals, effect sizes, degrees of freedom and *P* value noted<br>*Give P values as exact values whenever suitable.*
☒	☐	For Bayesian analysis, information on the choice of priors and Markov chain Monte Carlo settings
☒	☐	For hierarchical and complex designs, identification of the appropriate level for tests and full reporting of outcomes
☒	☐	Estimates of effect sizes (e.g. Cohen's *d*, Pearson's *r*), indicating how they were calculated

Our web collection on statistics for biologists contains articles on many of the points above.

Software and code

Policy information about availability of computer code

Data collection	Cell Profiler v3.1.9; TopSpin v3.5; Bio-Rad Image Lab v6.0.1; GROMACS v2018.2
Data analysis	ImageJ v1.52a; TopSpin v3.5; FoxTrot v2.0.2; VMD v1.9.3

For manuscripts utilizing custom algorithms or software that are central to the research but not yet described in published literature, software must be made available to editors and reviewers. We strongly encourage code deposition in a community repository (e.g. GitHub). See the Nature Research guidelines for submitting code & software for further information.

Data

Policy information about availability of data

All manuscripts must include a data availability statement. This statement should provide the following information, where applicable:

- Accession codes, unique identifiers, or web links for publicly available datasets
- A list of figures that have associated raw data
- A description of any restrictions on data availability

Provide your data availability statement here.

Field-specific reporting

Life sciences study design

All studies must disclose on these points even when the disclosure is negative.

Sample size	For Microtubule bench experiments, the length analyzed along the microtubule network was at least 0.5 mm for each condition. The number of clusters was determined and indicated. Paired two-sample Kolmogorov-Smirnov - and t-tests were used to test the significance. Four biological replicates were performed for each condition and values were then processed as described by Maucuer et al. J Cell Sci (2018).<br>For Stress granule experiments, cell cytoplasm and nucleus were detected automatically by using Cell Profiler. Values were then analyzed in order to statistically distinguish the distribution of YB-1 or Lin28 protein in cells. The cell number chosen provides a sufficiently good statistical significance regarding the changes analyzed in this study.<br>For Molecular Dynamics simulations, the sample size for prediction was based on 200 ns of NPT production run (except for Lin28:RRM1-TDP43:Lin28:RNA trimer complex where we ran only 10 ns of MD, which was sufficient to observe the destabilization of the complex)
Data exclusions	NO
Replication	Experiments were repeated in two or three independent replicates, as indicated in figure legends or in the online methods to reliably support conclusions stated in the manuscript.
Randomization	No data ramdomization was performed for any of experiments cited in this study.
Blinding	No blinding was applied in this study.

Reporting for specific materials, systems and methods

We require information from authors about some types of materials, experimental systems and methods used in many studies. Here, indicate whether each material, system or method listed is relevant to your study. If you are not sure if a list item applies to your research, read the appropriate section before selecting a response.

Materials & experimental systems

n/a	Involved in the study
☐	☐ Antibodies
☐	☒ Eukaryotic cell lines
☒	☐ Palaeontology and archaeology
☒	☐ Animals and other organisms
☒	☐ Human research participants
☒	☐ Clinical data
☒	☐ Dual use research of concern

Methods

n/a	Involved in the study
☒	☐ ChIP-seq
☒	☐ Flow cytometry
☒	☐ MRI-based neuroimaging

Antibodies

Antibodies used	Information about the commercial antibodies are provided in the manuscript. Oly YB-1 antibody are home made.
Validation	The home made primary antibody, Anri-YB-1, has been tested by siRNA (Figure S7B)

Eukaryotic cell lines

Policy information about cell lines

Cell line source(s)	NCS-34, HeLa cells, HEK 293 (ATTC)
Authentication	None
Mycoplasma contamination	HeLa cells and HEK 293 celles were tested negative for mycoplasma contamination.
Commonly misidentified lines<br>(See ICLAC register)	The cell line used in this study is not listed in the ICLAC database.
